# Recruitment of People with Cognitive Impairment and Caregivers for a Randomized Clinical Trial

**DOI:** 10.1093/geroni/igaf122.3468

**Published:** 2025-12-31

**Authors:** Pei-Shiun Chang, Amy Katz, Richard Passey, Ziyi Yang, Sujuan Gao, Liana Apostolova, Yvonne Lu

**Affiliations:** Indiana University Bloomington, Bloomington, Indiana, United States; Indiana University School of Nursing, Indianapolis, Indiana, United States; Indiana University School of Medicine, Indianapolis, Indiana, United States; Indiana University School of Medicine, Indianapolis, Indiana, United States; Indiana University School of Medicine, Indianapolis, Indiana, United States; Indiana University, Indianapolis, Indiana, United States; Indiana University School of Nursing, Indianapolis, Indiana, United States

## Abstract

Recruiting older people with cognitive impairment (PwCI) and their caregivers (CG) for research studies is an ongoing challenge, which was magnified by the onset of COVID-19. This study describes the unique recruitment of PwCI-CG dyads for a Randomized Control Trial using a tailored Daily Engagement in Meaningful Activity (DEMA) intervention to improve the quality of life for both PwCI and their CGs. Instead of traditional in-person recruitment, telephone-based recruitment was a driving factor for successfully contacting participants. Using a state-wide healthcare system database, we identified 4133 potential participants who met study criteria and could identify a primary caregiver. By mailing study information to potential PwCI-CG dyads, we observed a significant increase in initial contact, returned calls, and general interest in study participating. In-person recruitment, direct referrals from physicians in the clinic setting, and partnered local agencies were among the least successful strategies for establishing initial contact and recruiting dyads. After implementing new recruitment strategies, we recruited 152 dyads in 16 months (mean = 10 dyads/month). We found that establishing a monthly recruitment goal was a beneficial strategy. The healthcare system database, followed by an organizational registry, demonstrated higher rates of making initial contact and reaching eligible dyads (46% and 37%, respectively). Specific dyadic recruitment challenges included finding a suitable CG to participate and the extended follow-up timeframe from initial contact to screening and enrollment. There is a growing need to better understand successful strategies for didactic recruitment, particularly among older PwCI and their CGs.

